# Commensal bacteria and cancer immunotherapy: strategy and opportunity

**DOI:** 10.1093/lifemedi/lnad024

**Published:** 2023-06-23

**Authors:** Xusheng Zhang, Xiwen Qin, Shuo Wang

**Affiliations:** CAS Key Laboratory of Pathogen Microbiology and Immunology, Institute of Microbiology, Chinese Academy of Sciences, Beijing 100101, China; University of Chinese Academy of Sciences, Beijing 100049, China; Division of Infectious Diseases, Department of Medicine, Washington University School of Medicine, Saint Louis, MO 63110, USA; CAS Key Laboratory of Pathogen Microbiology and Immunology, Institute of Microbiology, Chinese Academy of Sciences, Beijing 100101, China; University of Chinese Academy of Sciences, Beijing 100049, China

The development and progression of cancer are intimately linked with the tissue microenvironment, specifically the interaction between the immune system, metabolites, and microbiome. The relationship between cancer and microbiota is complex and bidirectional, with changes in the microbial community serving as crucial factors that influence cancer progression or as a consequence of alterations in the tumor microenvironment. The connection between cancer and microorganisms has been established, with up to 20% of global cancer estimated to be caused by microorganisms such as *Helicobacter pylori*, *Fusobacterium nucleatum*, and other microorganisms [1]. Additionally, the human body harbors various commensal bacteria that form the human microbiome. Commensal microorganisms play a significant role in promoting host health by inhibiting the growth of pathogenic organisms, producing beneficial metabolites, and aiding in the digestion of nutrients. However, existing research suggests that commensal microorganisms may increase susceptibility to certain cancers and affect the response and prognosis of cancer treatment [1]. As a result, combining microbiota interventions with other cancer treatments has emerged as a novel strategy in cancer therapy.

**Figure 1.**
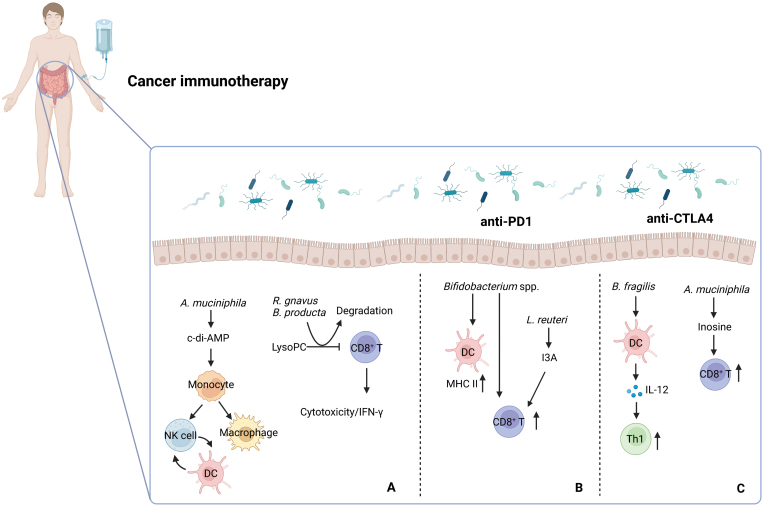
The influence of the gut microbiota on cancer immunotherapy. (A) *Akkermansia muciniphila* (*A. muciniphila*) produces c-di-AMP and activates monocytes, which promotes macrophage polarization and natural killer (NK) cell-dendritic cell (DC) crosstalk. Lachnospiraceae family bacteria *Ruminococcus gnavus* (*R. gnavus*) and *Blautia producta* (*B. producta*) degrade lysophosphatidylcholine (LysoPC), activate CD8^+^ T cells, and promote cancer immunosurveillance. (B) *Bifidobacterium* spp. and *Lactobacillus reuteri* (*L. reuteri*) activate CD8^+^ T cell and promote efficacy of anti-PD1 therapy. *Bifidobacterium* spp. increase MHC II^high^ DCs and activate CD8^+^ T cells, which enhance response to anti-PD1 therapy. *L. reuteri* produces AhR agonist indole-3-aldehyde (I3A), and promotes the efficacy of anti-PD1 immunotherapy. (C) *Bacteroides fragilis* (*B. fragilis*) and *Akkermansia muciniphila* (*A. muciniphila*) promote efficacy of anti-CTLA4 therapy. Nonenterotoxin-producing strains of *B. fragilis* prime IL-12 production of bone marrow-derived DCs and induce T helper 1 (Th1) immune responses. *A. muciniphila* produces inosine which binds to adenosine A2A receptor (A2AR) of CD8^+^ T cells, promotes the expression of IFN-γ of CD8^+^ T cells, and improves the efficacy of anti-­CTLA4 immunotherapy. NK cell, natural killer cell; DC, dendritic cell; LysoPC, lysophosphatidylcholine; IFN, interferon; MHC II, major histocompatibility complex class II; I3A, indole-3-aldehyde; Th1, T helper 1.

The microbiota plays a vital role in training and developing the host’s innate and adaptive immune system. Commensal bacteria also regulate cancer immunotherapy based on modulating host immune function. Approximately 50% of plasma metabolites are estimated to derive from symbiotic bacteria, highlighting the critical role of the microbiota in host metabolism [1]. The microbiota can affect the activation of the host immune system by synthesizing metabolites that, in turn, can influence the development and progression of tumors [1]. Commensal flora has a dual effect on the immune system’s regulation and tumor development. On the one hand, commensal flora directly regulates the immune system and inhibits tumor development. On the other hand, commensal flora can be used simultaneously as tumor immunotherapy drugs to improve treatment outcomes. *Akkermansia muciniphila*, a common commensal probiotic, plays a key role in inhibiting cancer progression. Intratumoral *A. muciniphila* suppresses tumor growth by generating the interferon gene (STING) agonist c-di-AMP, inducing an increase in type I interferon (IFN-I) production of tumor-infiltrating monocytes, and promoting macrophage polarization and natural killer cell (NK)-dendritic cell (DC) crosstalk [2] (Fig. 1A). Adaptive immune cells are also regulated by microbiota. Recently, our group discovered that normal colon tissue exhibits high levels of Lachnospiraceae family bacteria (*Ruminococcus gnavus* and *Blautia producta*), which are capable of degrading lyso-glycerophospholipids in tissues, promoting the function of CD8^+^ T cells and immunosurveillance, and resulting in the suppression of colon tumors [3] (Fig. 1A). Moreover, Scharl’s group (University of Zurich, Switzerland) showed that a mix of four *Clostridiales* species including *Roseburia intestinalis*, *Eubacterium hallii*, *Faecalibacterium prausnitzii*, and *Anaerostipes caccae* possess antitumor activity by promoting infiltration and activation of CD8^+^ T cells in tumors. These commensal bacteria have the potential to be used as microbial drugs.

The microbiota plays a crucial role in modulating the immune system, and can influence the outcome of immunotherapy. After treatment with antitumor prophylactic agents such as metformin and apple procyanidins, the abundance of *A. muciniphila* increases and is associated with the synthesis of the metabolite inosine. Inosine produced by *A. muciniphila* can promote CD8^+^ T cell activation, thereby improving the effectiveness of immune checkpoint blockade (ICB) [4]. Recent study by Meisel’s group (University of Pittsburgh School of Medicine, USA) showed that *Lactobacillus reuteri*, can catabolize dietary tryptophan into indole-3-aldehyde (I3A) through aromatic amino acid aminotransferase. I3A is an aryl hydrocarbon receptor (AhR) agonist that activates CD8^+^ T cells and effectively inhibits tumor growth in combination with anti-PD1 treatment (Fig. 1B). *Bifidobacterium* spp. upregulate MHC II^high^ DCs and promote the function of cytotoxic T lymphocytes (CTLs), resulting in improved efficacy of anti-PD1 immunotherapy and patient survival [1]. Nonenterotoxin-producing strains of *Bacteroides fragilis* prime IL-12 production of bone marrow-derived DCs and activate Th1 cells, enhancing the efficacy of anti-CTLA4 immunotherapy [1] (Fig. 1C). These outcomes have been demonstrated in patients with metastatic melanoma. *A. muciniphila* also has anticancer properties and can enhance the efficacy of anti-CTLA4 immunotherapy through the synthesis of inosine and activation of CD8^+^ T cells [4] (Fig. 1C).

There is a significant potential to prevent or treat human cancer through the use of microbiota-based therapies. Numerous investigational microbiome medicines are currently undergoing regulatory processes. One such medicine is MRx0518 from 4D Pharma (Aberdeen, UK), a capsule containing a live biotherapeutic that combats solid-tumor cancers in combination with pembrolizumab, which is undergoing phase I/II clinical trials. Additionally, Vedanta Biosciences’ VE800 (USA), consisting of 11 clonal human commensal bacteria strains that potentiate the immune system against tumors and enhance the effects of ICB [5], is also in phase I/II clinical trials. Despite the microbiome’s anticancer potential, no products have been successfully licensed as microbiome medicines to date. Fecal microbial transplant (FMT) is another approach to using microbiota for disease treatment, which is now accepted as a treatment in clinical practice (with some restrictions). In a phase 1 clinical trial of FMT combined with anti-PD1 immunotherapy in patients with anti-PD1-refractory metastatic melanoma, treatment with FMT was associated with immune cell infiltration in the tumor microenvironment, suggesting the potential of FMT in cancer immunotherapy.

Immunotherapy has revolutionized cancer treatment, and the gut microbiome plays a crucial role in the therapeutic response. Various commensal bacteria and probiotics influence the immune response throughout cancer progression, making gut microbiome modulation a promising cancer immunotherapy approach. Most commensal bacteria with immunotherapy potential come from the normal human intestine, making them safer and potentially causing fewer side effects than chemical or antibody drugs. But we should also note that different types of commensal bacteria may also have different effects on the development of tumors. Jack’s group (from Massachusetts Institute of Technology, USA) showed that the commensal bacteria promote inflammation and tumor cell proliferation during the lung tumor progression. Therefore, identifying the function of different commensal bacteria and precisely using them in cancer therapy is one of the challenges ahead. This relies on the basic research, which requires us to study the mechanism of interaction between commensal bacteria and the host in more depth. Another challenge is how to standardize production and obtain drugs with consistent activity. This relies on the industry, and requires standardization of industrial production techniques for commensal bacteria.

In the future, microbial drugs may be used as early tumor treatments or as adjunctive drugs for advanced tumor treatment. In the early stages of tumor discovery, a microbial drug intervention may activate the body’s immune system and cause early tumor regression without surgery. In advanced tumor treatment, microbial drugs may regulate intestinal flora disorders and balance the excessive immune response caused by chemical or antibody drugs. We believe microbial drugs will have broad application prospects in the future.
